# Supplementary material for: Sequelae following an epidemic of meningococcal meningitis in Niger in 2022
Source: PLoS One. 2025 May 22;20(5):e0323223. doi: 10.1371/journal.pone.0323223 (PMC12097593; doi:10.1371/journal.pone.0323223)
Supplement: S1 Fig — NB: The red lines represent the epidemic threshold in a HA; the orange lines represent the alert threshold in a HA. (DOCX) [file pone.0323223.s001.docx]

# **S1 Figures: Epidemic curves in the 5 Health Areas that crossed the epidemic threshold during the epidemic.** NB: The red lines represent the epidemic threshold in a HA; the orange lines represent the alert threshold in a HA.
