# Supplementary material for: Sequelae following an epidemic of meningococcal meningitis in Niger in 2022
Source: PLoS One. 2025 May 22;20(5):e0323223. doi: 10.1371/journal.pone.0323223 (PMC12097593; doi:10.1371/journal.pone.0323223)
Supplement: Attachment — Submitted filename: Reviewer 3 Comments.docx [file pone.0323223.s003.docx]

**Reviewer #3 Comments of Revision 1of the manuscript**

General Comment

I reviewed Revision 1 of the manuscript, i.e., following review by Reviewers 1 and 2.

I must say that the manuscript is well written, and the work done feeds into many areas of the meningitis education, epidemiology and clinical aspects as mentioned by previous reviewers.

The context and content have been well presented and compliment the previous work done in the region as far as epidemics are concerned.

In addition to what have already been highlighted by Reviewers 1 and 2, I have the following few comments to be considered by the authors:

Line 24 – 26: Please break the first sentence into 2 parts – seems too long and contain 2 main info.

Line 71 – 75: are you able to state the current treatment guidelines in Niger for treating sequelae following meningococcal meningitis. If Corticosteroids are prescribed by some doctors, do they do so by their own discretion?

Line 80 – 82: is/was there a follow up to ascertain the effectiveness of the vaccination campaign in this cohort, especially in those under 5 years?

Lines 93 and 97: “Suspected case” rather?

Line 114: Consider “Verbally” informed consent……

Line 125 – 126: please change to **16th** and **30th** November 2022; and similar references throughout the script.

Line 172 – 174: Can you please add the complete in-text reference, year of publication of these ethical guidelines and owners/authors.

Line 193: 1001, comma not needed in the figure.

Line 297: Please delete “during the epidemic.”
